# The Interplay Between Chromatin Architecture and Lineage-Specific Transcription Factors and the Regulation of *Rag* Gene Expression

**DOI:** 10.3389/fimmu.2021.659761

**Published:** 2021-03-16

**Authors:** Kazuko Miyazaki, Masaki Miyazaki

**Affiliations:** Laboratory of Immunology, Institute for Frontier Life and Medial Sciences, Kyoto University, Kyoto, Japan

**Keywords:** lineage-specific transcription factor, chromatin architecture, enhancer, *Rag1* and *Rag2* gene, super-enhancer, 3D genome organization

## Abstract

Cell type-specific gene expression is driven through the interplay between lineage-specific transcription factors (TFs) and the chromatin architecture, such as topologically associating domains (TADs), and enhancer-promoter interactions. To elucidate the molecular mechanisms of the cell fate decisions and cell type-specific functions, it is important to understand the interplay between chromatin architectures and TFs. Among enhancers, super-enhancers (SEs) play key roles in establishing cell identity. Adaptive immunity depends on the RAG-mediated assembly of antigen recognition receptors. Hence, regulation of the *Rag1* and *Rag2* (*Rag1/2*) genes is a hallmark of adaptive lymphoid lineage commitment. Here, we review the current knowledge of 3D genome organization, SE formation, and *Rag1/2* gene regulation during B cell and T cell differentiation.

## Introduction

Adaptive immunity relies on the assembly of antigen recognition receptor genes (T cell receptor (*TCR*) and immunoglobulin (*Ig*) genes) from arrays of variable (V), diversity (D), and joining (J) gene segments that enable the receptors to recognize highly diverse antigens and to invoke antigen-specific immune responses. This process is initiated by a protein complex composed of recombination-activating gene 1 (Rag1) and Rag2, which can recognize and cleave recombination signal sequences (RSSs) flanking the TCR and Ig V, D, J gene segments (1, 2). After T cell lineage commitment is induced by Notch signaling in the thymus, TCRβ V(D)J rearrangement is initiated at the immature CD4^–^CD8^–^ (double negative, DN) DN2-3 stages. Following the β-selection of the TCR, DN cells differentiate into CD4^+^CD8^+^ (double positive, DP) cells, and VJ rearrangement of TCRα is initiated. Similarly, V(D)J recombinations of Ig heavy-chain (*Igh*) and light-chain occurs in B cell progenitors (pro-B cells) and precursors (pre-B cells), respectively (3, 4). The recombination of TCRγδ occurs concurrently with TCRβ D-J recombination at the DN2 stage, preceding TCRβ V-to-DJ recombination (5). Because RAG-mediated DNA cleavage can lead to oncogenic translocations, *Rag1* and *Rag2* (*Rag1/2*) expression is stringently controlled in a highly cell type-specific manner; as are expressed only in T and B cell progenitors and precursors (DN3 and DP thymocytes, pro-B and pre-B cells) (6–9). Therefore, *Rag1/2* gene expression typically reflects the cell fate decision toward adaptive immune cells.

Common lymphoid progenitors (CLPs) in the bone marrow (BM) give rise to B cells, T cells, dendritic cells (DCs) and innate lymphoid cells (ILCs), including natural killer (NK) cells. In addition to environmental signals, the adaptive lymphoid cell lineage is specified by the sequential expression of an ensemble of transcription factors (TFs): E2A, Ebf1, Foxo1, and Pax5 for B cell development and E2A/HEB, Gata3, Tcf1, Bcl11b, Runx, Ikaros, and Pu.1 for T cell development (3, 10–12). However, ILCs and T lineage cells express a common set of TFs, such as Gata3, Tcf1, Bcl11b, and Runx, consistent with their similar expression of effector cytokines (13–22). What TFs drive adaptive lymphoid lineages and differences between T cells and ILCs? Namely, E2A and HEB establish T cell identity and suppress the development of thymic ILCs by modulating the repertoires of enhancers, while Pax5 and Ebf1 are required for B cell lineage commitment because they repress genes leading to alternative cell fates for T cells and ILCs (23, 24). Because the biggest difference between T cells and ILCs is RAG-mediated TCR recombination, adaptive lymphoid lineage-specific TFs, which suppress the ILC program, are thought to regulate *Rag1/2* genes to make differences between adaptive and innate lymphocytes. Most adaptive lymphocyte development trajectories require regulation by members of the helix-loop-helix families, such as E proteins (E2A, HEB, and E2-2) and Id proteins (Id1-4) (25). The E protein binds to the E-box motif (CANNTG) within enhancer regions of its target genes, the DNA-binding activity of the E protein is antagonized by Id proteins, and Id2 is particularly important for ILC, NK, and LTi cell development (26–28). Therefore, it is proposed that the E-Id protein axis specifies innate and adaptive lymphoid cell fates.

Gene promoters are genetic regulatory elements that provide platforms for TFs to bind and regulate gene expression. However, the regulation of promoter regions is generally insufficient for the cell type-specific regulation of genes that are required for cell functions. Therefore, many genomes contain numerous regulatory elements known as enhancers (29). Lineage-specific TFs alter gene expression patterns by binding to specific DNA sequences within cis-regulatory elements (CREs), including promoters and enhancers. These factors can also change chromatin architecture to determine lineage cell fate and to constrain the development of other lineages. Therefore, the establishment and maintenance of cell type-specific gene expression programs are the result of the interplay between lineage-specific TFs and chromatin architecture, and this interplay can function as a barrier, primer, optimizer, or facilitator to control cell fate. Precisely characterizing this interplay will have profound implications for understanding the development of not only cells but also diseases, such as cancer (30, 31).

Recent studies have identified a novel class of enhancers termed super-enhancers (SEs). An essential feature of SEs is their ability to control genes that have prominent roles in cell type-specific functions, thereby establishing cell identity (32, 33). A SE displays a property of highly cooperative interactions with numerous TFs, mediators and RNA polymerases (34–36). Taking these characteristics into account, many lineage-specific TFs are thought to be associated with each other to form adaptive lymphocyte-specific SEs in *Rag1/2* gene loci in developing T and B cells.

Many studies examining the role of chromatin architectures have focused on the recombination of *TCR* and *Ig* loci, and many excellent review papers have been published about the importance of three-dimensional (3D) genome organization in antigen receptor loci. Therefore, in this review paper, we focus on adaptive lymphoid cell-specific gene regulation that does not involve *TCR* or *Ig* genes. Here, we review the mechanisms of 3D genome organization and SE formation by cell type-specific TFs and explain how cell type-specific expression of the *Rag1/2* is mechanistically regulated by CREs, key TFs, and the chromatin architecture. In particular, we focus on the regulation of *Rag1/2* genes in developing T and B cells because this locus serves as a template that can be extrapolated to other lineage-specific regulatory events.

## 3D Genome Conformation and Super-Enhancers

**Figure 1:**
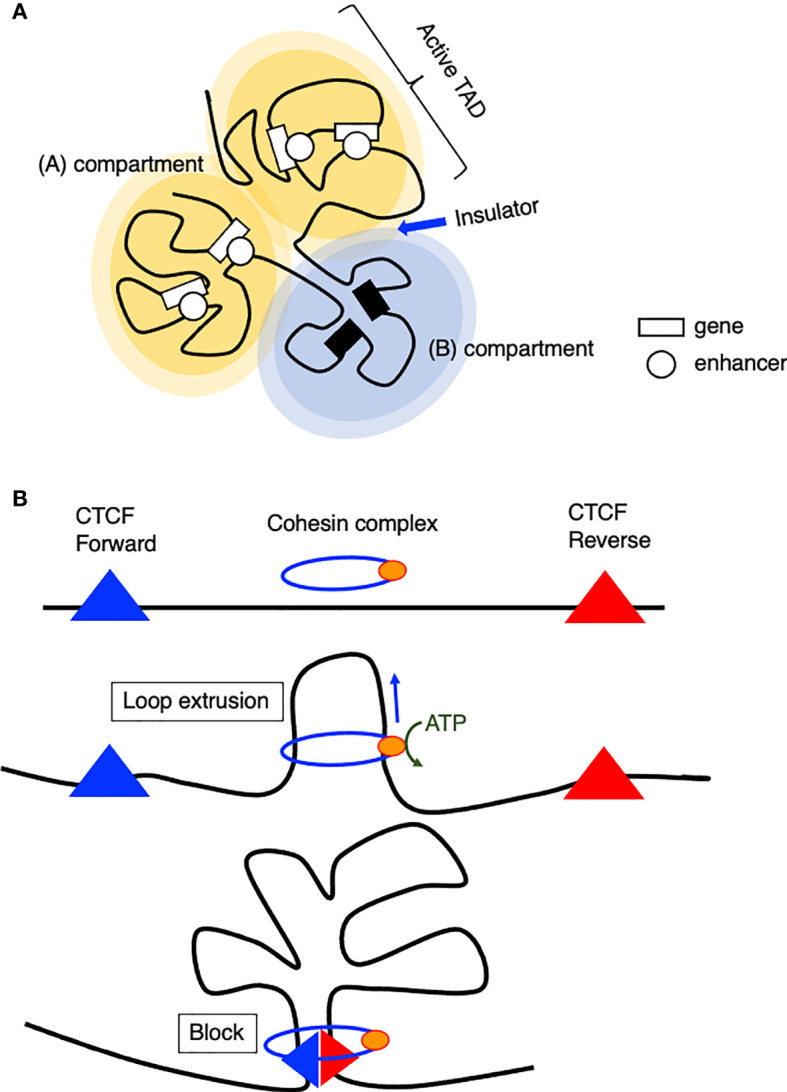
Model of chromatin organization. **(A)** Spatial chromosome folding in the nucleus separates the genome into (A, B) compartments (A) active compartment, (B): repressive compartment). (A, B) compartments are composed of smaller domains called topological associating domains (TADs), which preferentially interact with themselves rather than with other regions. **(B)** Model of loop extrusion realized by cohesin ATPase motor activity.

The chromatin of the mammalian genome is hierarchically organized into two large compartments, known as transcriptionally permissive (A) and repressive (B) compartments, and smaller domains called topologically associating domains (TADs) (**Figures 1A, B**) (37–39). TADs contain smaller sub-TADs characterized by higher interaction frequencies. The boundaries of TADs are enriched with insulator binding protein CCCTC-binding factor (CTCF) and highly transcribed housekeeping genes, which play important roles in establishing TAD structure, and the disruption of TAD structures or the dysfunction of these insulators results in the pathogenic reconfiguration of enhancer-promoter interactions (38, 40–42). In the (A) compartment, genes are generally transcribed, and active histone modifications are observed. In contrast, the (B) compartment primarily contains inactive genes and is associated with repressive histone modifications (37). Conspicuously, the *Ebf1* gene locus switches from the (B) to the (A) compartment upon B cell lineage commitment (43). Furthermore, chromatin interactions both within and between domains change dramatically, showing extensive (A)/(B) compartment switching during the differentiation of human embryonic stem (ES) cells, while the positioning of TADs remains stable between cell types (44). Interestingly, once a particular cell type is established, extracellular signaling–responsive enhancers are in contact with their target promoter regions even before activation, and looping interactions are largely unchanged after enhancer activation and target gene expression (45).

How are TADs organized? The loop extrusion model suggests that the cohesin complex progressively extrudes chromatin until two relevant CTCF molecules encounter and blocks the extruding chromatin at a convergent head-to-head position (46, 47). This chromatin extrusion is mediated by the ATPase activity of the Smc protein, a component of the cohesin or condensin complex (48, 49), extruding tens of kilobase pairs of DNA at a speed of up to 1500 base pairs per second *in vitro* (50). In addition, this extrusion is asymmetric, which indicates that one site is anchored onto DNA and reels in the chromatin on one side. According to Hi-C data, a loop anchor appears as stripes because it interacts with entire domains, and this stripe- or super-anchor often tethers a SE to a cognate promoter (**Figure 1B**) (49, 51).

**Figure 2:**
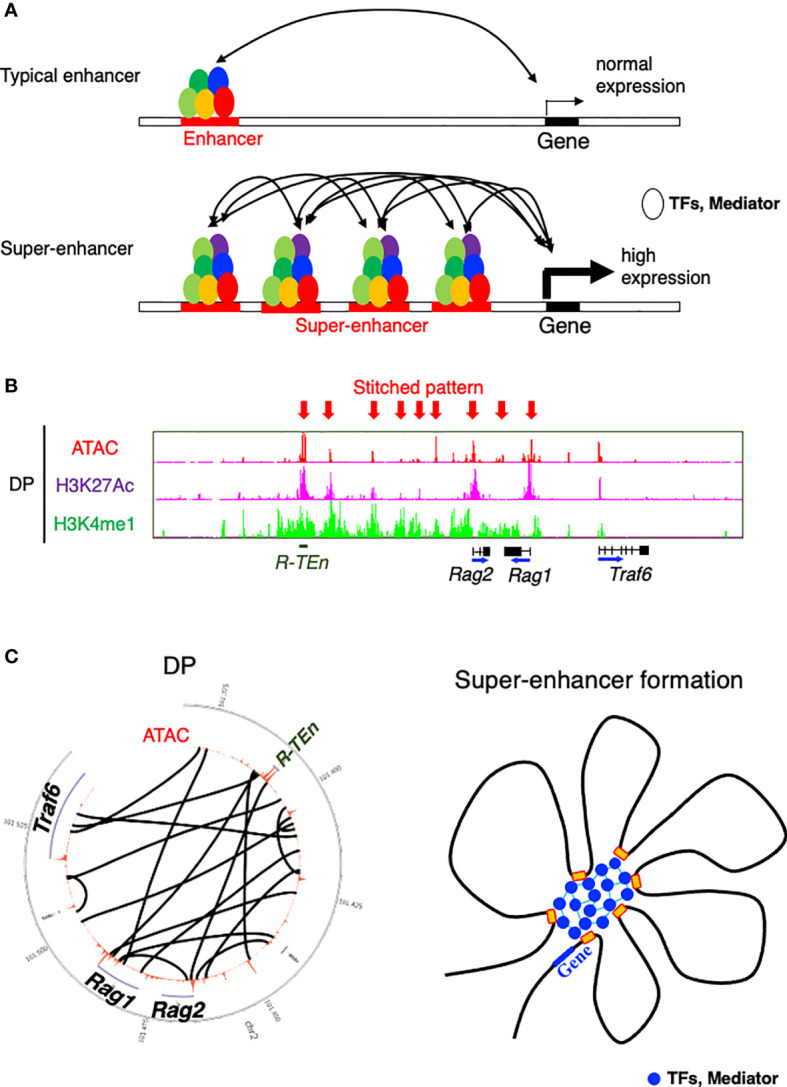
Model of a super-enhancer. **(A)** Comparison between typical enhancers and super-enhancers (SEs). SEs are occupied by a highly dense population of transcription factors (TFs) and transcriptional mediators. DNA binding regions within SE cooperatively bind to each other, which contributes to the high expression of target genes (36). **(B)** ChIP-seq [H3K27 acetylation (active region) and H3K4 monomethylation (enhancer)] and the ATAC-seq profile of the *Rag* gene SE (52). Arrows indicate a stitched pattern of enhancers. **(C)** Circos diagram representing genomic interactions (black lines) across *Rag* gene locus in DP cells (left) (52) and model showing TFs and mediators forming phase-separated condensates at SE regions (36).

SEs are defined as being highly enriched with transcriptional coactivators and TFs and a high level of histone acetylation (32, 33). SEs exhibit high cooperativity between cell type-specific TFs, mediators, P300, Brd4, and RNA polymerase II, appearing at approximately 10-fold greater density than typical enhancers and contributing to higher transcriptional output (**Figure 2A**) (36). SEs span large genomic regions, and the clusters of enhancers within SEs are in close physical contact with each other and with promoter regions (**Figures 2B, C**). SEs are often formed near loci of genes that define cell identity and can be formed as a consequence of the binding of a single TF, a mechanism that makes the SEs vulnerable to perturbation of key components (34–36). On the basis of these features of SEs, a “phase separation model” has been proposed to explain the mechanism by which transcriptional regulators in high concentrations at SEs are associated with higher levels of transcription (transcriptional bursting) and the vulnerability of the SE structure in the absence of its key component (36, 53). TFs control gene expression by binding to DNA in enhancer or promoter regions. While the structure and function of the DNA-binding domains of TFs have been extensively studied, comparatively little is understood about the function of activation domains or other regions of TFs (36). These regions are enriched with intrinsically disordered regions that form liquid-liquid phase-separated droplets in combination with mediator coactivators Med1 and Brd4 at SE regions to compartmentalize and concentrate the transcription apparatus for the induction of robust transcription of cell identity genes (53). In line with this mechanism, the sol-gel phase transition of chromatin enables both rapid and ordered reassembly of the IgH locus for V-D-J recombination in developing B cells, which may be associated with the TF assembly of E2A, PU.1, Foxo1, and Pax5 (54). Therefore, the establishment of cell identity and lineage-specific gene expression programs requires the interplay of lineage-specific TFs and the chromatin architectures, as seen in SE formation.

## The Regulation of *Rag1/2* Expression by cis-Regulatory Elements (CREs) in Developing B Cells and T Cells

### *Rag1/2* Gene Enhancers

RAG expression is restricted to developing T and B lymphocytes. In both lineages, there are two waves of RAG expression (8). The first wave of RAG expression is required for the assembly of *Ig* heavy chain (IgH) and *TCRβ* genes in pro-B and pro-T cells, respectively. In developing thymocytes, RAG expression also catalyzes TCRγ and TCRδ gene rearrangement to become γδT cells in early DN populations. *Rag* expression is transiently downregulated in cells that successfully rearrange *Igh* or *TCRβ* during the developmental transition from progenitor to precursor (pre-B or pre-T cells). In pre-B and pre-T cells, *Rag* genes are re-expressed leading to the assembly of Ig light (*IgL*) chain and *TCRα* genes, respectively. Following successful recombination of *IgL* or *TCRα* and assembly of *Ig* or *TCRαβ*, *Rag* gene expression is suppressed in mature B and T cells. Loss of *Rag* gene expression results in developmental arrest at the progenitor stages of T cell and B cell development (55, 56), while persistent *Rag* expression causes aberrant thymic development and profound immunodeficiency (57). Therefore, *Rag* expression is tightly regulated during adaptive lymphocyte development. Both *in vivo* and *in vitro* studies have attempted to identify the CREs for *Rag1/2* expression (7). Interestingly, while both B and T cells require *Rag1/2* expression for antigen receptor gene recombination, the *Rag* enhancers in B and T cells differ. In B cells, the deletion of *Erag*, which is 23 kb upstream of the *Rag2* promoter, resulted in a significant reduction in *Rag1/2* expression and a partial block at the transition from pro-B to pre-B cells, but did not affect thymocyte development in mice (58). It has been reported that the *Erag* enhancer is positively regulated by FOXO1 and negatively regulated by Gfi1b, Ebf1, and c-Myb (59–62). In contrast, an anti-silencer element (*ASE*), which is 8 kb in length and located 73 kb upstream of the *Rag2* promoter, is shown to be required for *Rag1/2* expression in DN3 and DP cells but not in B cells (63). Since *Erag* and *ASE* deletions resulted in the partial loss of RAG activity, additional CREs and their cognate binding factors are likely necessary (7). To my knowledge, there has been no report regarding *Rag1/2* expression with respect to TCRγδ gene recombination. Additionally, how enhancers regulates *Rag1* and *Rag2* in coordinate and how *Rag1/2* expression is tightly restrained in innate immune cells will be explored in a later section.

**Figure 3:**
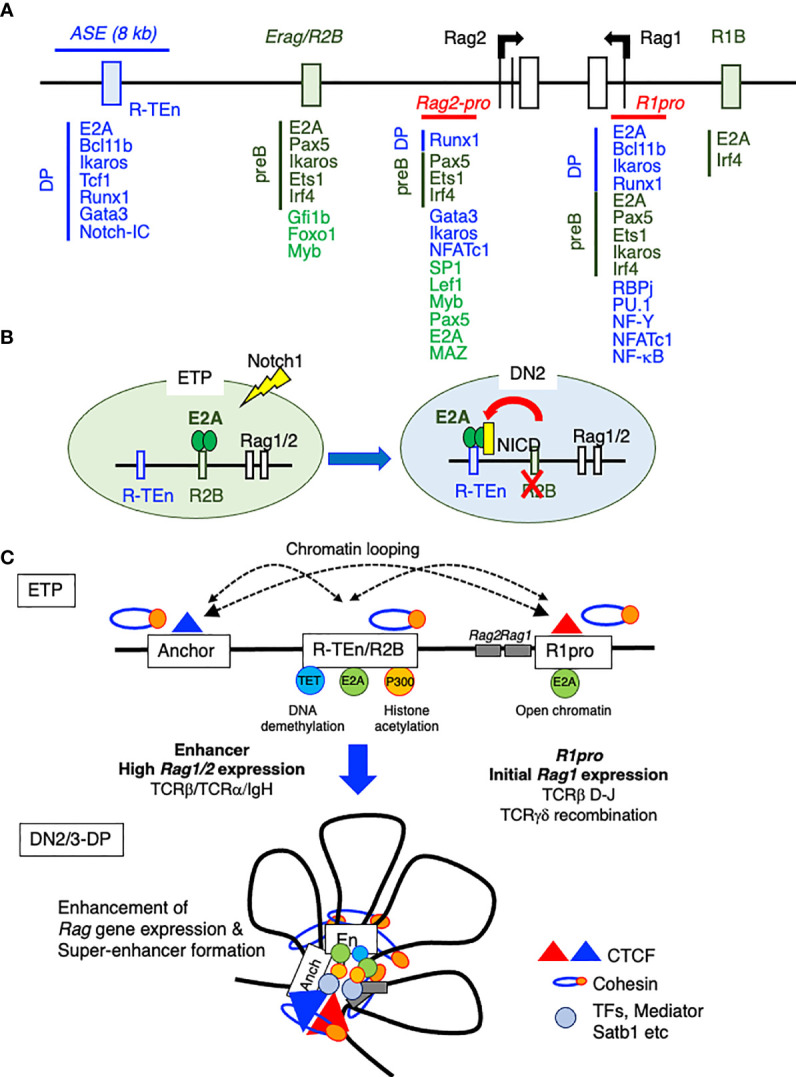
The *Rag* gene locus and cis-regulatory elements. **(A)** A schematic diagram of the *Rag* gene locus and cis-regulatory elements in T and B cell lineages. The green boxes indicate B cell-specific enhancer regions (*R1B* and *Erag/R2B*) and transcription factor (TF) binding in proB cells. The blue line and box indicate the T cell-specific enhancer region (*ASE* and *R-TEn*) and TF binding in DP cells. The red line indicates the common region between B and T cells (*R1pro*). **(B)** The recruitment of E2A to the T cell-specific enhancer region (*R-TEn*) upon T cell lineage commitment. **(C)** Model depicting the stepwise establishment of an adaptive lymphocyte-specific super-enhancer for *Rag1/2* expression mediated by E2A.

The Krangel group reported that *ASE* directly interacts with the *Rag1* and *Rag2* promoters and that the chromatin organizer Satb1 binds to this *ASE* to promote optimal *Rag1/2* gene expression through *Rag* gene locus organization in DP thymocytes, as proven by chromosome conformation capture (3C) assay (64). In addition, by using the VL3-3M2DP thymocyte cell line, this group demonstrated that the *ASE* requires Gata3 and E2A, while the *Rag1* promoter relies on Runx1 and E2A as critical regulators. Together, the *ASE* and *Rag1* promoter framework functions as a chromatin hub (65). Recently, we have identified cell type-specific CREs for *Rag1/2* expression in B and T cells using E2A ChIP-seq and ATAC-seq data, because E2A specifies adaptive lymphocyte and is the driver of differences in enhancer repertoires in T cells and ILCs (24, 52). We mapped E2A binding to one T cell-specific *Rag* gene enhancer [*Rag-*T cell enhancer (*R-TEn*) included in the 8-kb *ASE*] and to two B cell–specific *Rag* gene enhancers [*Rag* B cell enhancer 1/2; *R1B* (5-kb upstream of the *Rag1* promoter) and *R2B* (partially overlapping with *Erag*)] (**Figure 3A**) (52). *R-TEn* and *R1B/R2B* loop uniquely to the *Rag1/2* promoter regions and form different genome structures. In addition to E2A, T cell- or B cell-specific TFs (T cell: Bcl11b, Tcf1, Runx1, Ikaros, and Gata3; B cell: Pax5, Ets1, Ikaros, and Irf4) bind to these enhancer regions (**Figure 3A**). Furthermore, the *R2B* and *Rag1* gene promoter region (*R1pro*) in pre-B cells, and *R-TEn* along with the *Rag1/2* gene cluster in DP cells are SEs according to an analysis of histone acetylation (H3K27Ac) (52). Consistent with this finding, the *Rag* gene locus and its enhancers show elaborate chromatin interactions and are located in the active developing T cell- and B cell-specific sub-TAD (52). Furthermore, *R-TEn* deletion in mice specifically blocked thymocyte development during the β-selection of DN3 cells and positive-selection of DP cells, as seen in *ASE* deletion mice (52, 63). In addition, mice with *R1B/R2B* double deletions exhibited a severe developmental block at the pro-B stage, whereas the single deletion of either *R1B* or *R2B* led to mild or moderate impairments in B cell development. This outcome is comparable to that observed in *Erag* deficient mice, suggesting enhancer redundancy in *R1B* and *R2B* (7, 52, 58). These developmental defects in enhancer-deleted mouse lines were caused by failed *Rag1/2* gene expression and TCRαβ or IgH recombination. Interestingly, these cell type-specific enhancers are required not only for enhancer-promoter interactions, but also for other chromatin interactions across the *Rag* gene locus.

### *Rag1* and *Rag2* Promoters

It has been reported that T and B cell-specific TFs bind to *Rag1* and *Rag2* promoter regions (reviewed in (7)), and we also analyzed the occupancies of TFs in pre-B and pre-T cells (52). In DP cells, Bcl11b, Ikaros, NF-Y, NFATc1, and Runx1 are recruited to the *Rag1-*promoter region, while Pax5, Ets1, Ikaros, and Irf4 bind to this promoter in pre-B cells (7, 52). Runx1 in DP cells, and Pax5 and Irf4 in pre-B cells, but not other TFs, appear to bind to *Rag2* promoter regions in ChIP-seq analysis (7, 52). ChIP-seq data of Myb, Lef1, NFAT1c, and SP1 in pro-T and pro-B and pre-T and B cells are needed for further analysis. Interestingly, we found common E2A-binding sites in the *Rag1* gene promoter region (*R1pro*), but distinct E2A-binding sites in the *Rag2* promoter region in pro-T and pro-B cells (52). This finding is consistent with the differing regulation mechanism of the *Rag2* promoter in T and B cells (66, 67). To clarify the *R1pro* activity regulated by E2A, E-box motif mutations in *Rag1* promoter region were generated (*R1pro*-mut) to prevent the loss of other activities of the *Rag1* promoter. In addition, by generating E-box mutations in *R1pro*, the protein level of E2A and the binding of other TFs to this region were not affected. Blocking E protein binding to the *R1pro* region (*R1pro*-mut) resulted in the total loss of *Rag1* expression without affecting *Rag2* expression or enhancer activities in both pro-T cells and pro-B cells, causing developmental arrest at the T cell and B cell progenitor stages (52). This outcome suggests that *Rag1* promoter activity is commonly regulated by E protein binding in the adaptive lymphoid lineage. The regulation of *Rag2* promoter activity seems to be more complicated. It has been reported that two regions of *Rag2* promoter regions (D3 and Ep) are DNaseI hypersensitive sites to which C/EBP and NFKB bind to play important roles in the regulation of *Rag2* expression (7, 66–68). In addition, the Pax5/c-Myb/Lef1 protein complex in pre-B and Gata3 in T cells bind to *Rag2* promoter region to activate *Rag2* expression (69, 70). Although the expression of *Rag1*, but not *Rag2*, is severely affected in pro-T and pro-B cells derived from *R1pro-mut* mice, *Rag2* promoter CREs seem to regulate both the *Rag1* and *Rag2* genes in coordination (66). Therefore, it remains unknown whether *Rag2* promoter activity *in vivo* is independent similar to *R1pro*.

### Development and Evolution of *Rag* Gene CREs

How do B and T cells utilize the distinct CREs of *Rag* gene expression? According to a chromatin accessibility analysis, the *R2B* region first becomes accessible in the CLP stages and stays open until the pre-B cell stage. Interestingly, *R2B* is accessible at the uncommitted DN1 stage in the thymus but becomes inaccessible at the DN2a commitment stage. At the same time, *R-TEn* is closed in DN1 cells but open in DN2a cells, indicating mutual exclusivity of enhancer engagement between *R2B* and *R-TEn* after adaptive lymphoid lineage commitment. How is this sequential enhancer activity re-established? Because Notch1-Delta-like 4 (DLL4) signaling and E proteins are critically required for T cell lineage commitment and the suppression of ILC development in the thymus (24, 71, 72), Notch-RBP-J machinery possibly alters the binding of E-proteins to both *R2B* and *R-TEn* and makes *R-TEn* accessible (**Figure 3B**). This speculation is in line with the fact that CpG DNA in *R-TEn* is hypermethylated in pro-B and pre-B cells and *R1B/R2B* are hypomethylated in DN3 and DP cells, indicating that *R1B/R2B* used to be open in the T cell lineage. Notably, *R-TEn* is never open in the B cell lineage, and the demethylation of CpG islands is correlated with E2A binding. These results are consistent with a previous report showing the functional and physical interaction between E2A and TET proteins in developing B cells (73). We therefore speculate that *R1B* and *R2B* are primitive enhancers of *Rag1/2* expression in the lymphoid lineage and that *R-TEn*, a target of Notch signaling, was acquired later in evolution, after thymus anlage development in vertebrates. As *Dll4* is a direct target of Foxn1 and Foxn4 in thymic epithelial cells (TECs), it is important for studies of adaptive immunity to understand how the network of Foxn1/2-Dll4 in TECs (Notch ligand expression) and the Notch/E2A-*Rag* enhancer in progenitor T cells (interaction between Notch signaling and TF) were acquired during thymopoiesis evolution and confer the T cell-specific *Rag* enhancer activity (74–78).

Notably, *Rag1/2* promoter regions are accessible at the progenitor stage (DN3 and pro-B cells) without enhancer activity, while the accessibilities at these promoters depend on the enhancer activity at the precursor stage (DP and pre-B cells), suggesting that enhancer activity maintains the open status of *Rag1* and *Rag2* promoters through rounds of cell division during the transition from DN to DP stages (52). Consistent with this supposition, the level of E2A occupancy at *R-TEn* does not decline after pre-TCR signaling, which likely maintains *R-TEn* activity (79). When DP and pre-B cells differentiate into CD4SP or CD8SP and IgM^+^ B cells, *R1pro, Rag2* promoter, *R-TEn*, and *R2B* are immediately close, indicating that TCR and IgH signaling suppress these CRE activities (52). Blocking E2A binding to *R-TEn* and *R1pro* CREs is sufficient to disrupt *Rag1/2* SE formation and totally eliminate *Rag1* promoter activity, respectively (52). These outcomes are in line with the disappearance of chromatin interactions in *Rag* gene loci in mature T and B cells, which express a low level of E2A and a high level of Id2 and Id3. In fact, *Id3* is first induced by pre-TCR signaling and is further upregulated by TCR signaling, and this TCR-Id3 pathway is also important for γδT cell development (79–82). *Id2* is upregulated during later positive selection of DP cells, and Id2 and Id3 cooperatively function during positive selection of DP cells (83, 84). Therefore, Id proteins induced by TCR signaling antagonize the E protein to abrogate *Rag* gene SE and *Rag1* expression, inhibiting the subsequent expression of *Rag1/2* in mature T and B cells. In line with this process, 3D genome structures in developing T and B cells show changes as they develop. An increasing number of chromatin interaction loops across the *Rag* gene loci are observed during T and B cell developmental progression from CLPs, and these loops are absent when the *Rag1/2* genes are not expressed in mature T and B cells. The timing of loop formation correlates strongly with changes in the PC1 component and TADs and Loop scores during T and B cell development (52).

Interestingly, chromatin accessibility in most hematopoietic cells (granulocytes, erythrocytes, dendritic cells, NK cells, ILC2s and ILC3s), *R-TEn* and *R2B* are seldomly accessible in cells other than developing T and B cells (ImmGen data; GSE100738) (**Supplementary Figure 1**), according to an analysis of published data (68). On the other hand, *R1B* is slightly open in these cells, which is consistent with the fact that *R1B* functions as an insulator to sequester *Rag1/2* away from active genes (more details are present in the subsequent section). In contrast, we observed clear accessibility at the Nad Wyraz Ciekawy (*NWC*) locus in most hematopoietic cells, but not T and B cells. *NWC* is the third evolutionarily conserved gene with unknown function in the *Rag* gene locus and is located within the *Rag2* gene intron (**Supplementary Figure 1**). *NWC* seems to be a negative regulator of *Rag2* because of the mutual exclusivity of *Rag2* and NWC expression; however, it remains unknown whether transcripts from *NWC* block *Rag2* gene expression (7, 85–87). Upon the ILC lineage commitment, the E2A protein is suppressed in the ILC precursor, while *Id2* is highly expressed and maintained throughout ILC lineage, suggesting that Id2 inhibits E2A-mediated *Rag* gene CRE activities in the ILC lineage (24, 88).

Enhancers play important roles in precise gene expression programs during development, and divergence in enhancer sequence and activity is thought to be an important mediators of inter- and intraspecies phenotypic variation (89). Regardless of the level of enhancer sequence conservation, enhancers identified in human ES cells drive cell type- and stage-specific expression when introduced to zebrafish embryos, suggesting the conservation of ancestral functions of TFs (90). Additionally, the evolution of body shape is thought to be tightly coupled to changes in enhancer sequences of vertebrates. For example, a snake-specific sequence alterations within an otherwise highly conserved long-range limb enhancer of Sonic Hedgehog are closely associated with morphological changes of the limb in snakes, demonstrating the critical roles of enhancers in morphological evolution (91). These previous reports suggest that the conservation of DNA sequences within enhancer regions among species reflects the evolution of *Rag* gene regulation. *Rag1/2* genes are conserved among jawed vertebrates, in which they contribute to a diverse repertoire of antibodies and T cell receptors. The generation of this diversity is a pivotal event in the evolution of the adaptive immune system of jawed vertebrates (6). Therefore, to understand the evolution of adaptive immunity, it is also important to investigate the conservation of DNA sequences in T cell- and B cell-specific enhancers. Interestingly, *R-TEn* and *R2B* are highly conserved among mammals, as well as most birds and reptiles, but not in amphibians or fish. Furthermore, conserved *R-TEn*, *R1B*, and *R2B* were found to harbor conserved E-boxes (52). These observations reveal the discordance in the evolutionary conservation of *Rag* genes and their regulatory elements among jawed vertebrates and suggest the possibility that there are various regulatory mechanisms in terrestrial animals, aquatic animals, and amphibians. Here, we propose that adaptive immunity in terrestrial animals is evolutionarily developed *via* the utilization of E protein activity to increase the expression of *Rag* genes, which enables receptors to recognize diverse antigens by RAG-mediated *TCR* and *Ig* gene recombination. Considering the evolution of the *Rag* gene, the ancestral *Rag* transposon (Transib) contained a core region of the *Rag1* precursor, and subsequently, the *Rag2*-like gene was acquired by Transib, leading to the emergence of the *Rag* transposon that gives rise to the *Rag* and *Rag-like* genes found in echinoderms, cephalochordates, and jawed vertebrates (6). Furthermore, recent excellent studies of ProtoRAG transposase illuminate the evolution of RAG transposons and V(D)J recombination for the development of the adaptive immunity system in the human body (92, 93).

## Organization of the 3D Genome Architecture Contributes to B Cell and T Cell Identity and *Rag* Gene Super-Enhancer Formation

Upon the cell lineage decision-making of multipotent cells, abrupt genomewide changes in chromatin accessibility, chromatin interaction and conformation, and transcriptome occur, and these concerted changes in chromatin architecture act as barriers to prevent cells from reversing or being redirected to other lineages (94). As Lin and Murre reported, in B cell development, CTCF occupancies are associated with intradomain interactions, whereas P300, E2A and PU.1 are associated with intra- and interdomain interactions that are developmentally regulated in pro-B cells. During B cell lineage commitment from the pre-pro-B to pro-B stages, *Ebf1* is sequestered at the nuclear lamina in the pre-pro-B cells, and following commitment, *Ebf1* and other gene loci switch compartments to establish new intra- and interdomain interactions associated with a B cell lineage-specific transcription signature (43, 95). Recently, a prion-like-domain in the C-terminal domain of EBF1 has been shown to have a liquid-liquid phase separation ability, which enables the recruitment of the RNA-binding protein FUS and chromatin remodeler Brg1, which facilitate chromatin opening and acts as pioneer factors to induce the formation of phase-separated SEs for promoting B cell identity gene expression (96). After the global network consisting of E2A, Ebf1, and Foxo1 establishes B cell identity, Ebf1 and Pax5 maintain the B cell signature throughout the changes in the global lineage-specific genome architecture (23, 95, 97).

Upon T lineage commitment, regulome and 3D genome architectures are reorganized by T cell-specific TFs, such as Bcl11b and Tcf1, and noncoding transcripts (*ThymoD*) at the Bcl11b enhancer (3, 94, 98–100). Likewise Ebf1 locus upon B cell lineage commitment, *ThymoD* transcripts orchestrate chromatin folding and compartmentalization by activating cohesin-dependent looping to translocate its enhancer from the nuclear lamina to the nuclear interior (98). In each scenario, enhancer activity mediated by lineage-specific TF binding appears to be the first step for chromatin compaction and compartment switching.

*Rag1/2* expression and chromatin accessibility of *R-TEn* were not affected in *Tcf1*-, *Bcl11b*-, or *ThymoD*-deficient thymocytes, as determined by examining in previously published data (52). Furthermore, the expression of *Rag1/2* was not reduced in *Runx1*-deficient pro-B cells (101). Upon the deletion of *Satb1* or cohesin (*Rad21*), histone acetylation (H3K27Ac) levels at the *R-TEn* and *Rag1* promoter regions remained high, although *Rag1/2* expression was reduced in *Satb1*-deficient DP cells (64, 102–104). On the other hand, E-protein deletion (*E2A* (*Tcf3*) *and HEB* (*Tcf12*)) in DP cells led to the downregulation of *Rag1/2* and a reduction in chromatin accessibility at the *R-TEn* and *Rag1/2* promoter regions (52, 105). Mutating the seven E-box motifs in the *R-TEn* region (*R-TEn-E-box-mutant*) reduced *Rag1/2* expression and blocked thymocyte development. These results are similar to the effects of deleting the entire *R-TEn* region. Furthermore, the *R-TEn-E-box-mutant* abolished the chromatin accessibility and SE formation throughout the entire *Rag* gene locus in DP cells, which was accompanied by a significant reduction in cohesin recruitment. Because the enhancer activity of *R-TEn* drives active sub-TAD formation, we considered how this active TAD is initiated after the enhancer is activated. For active TAD formation, CTCF and cohesin facilitate loop extrusion by using ATP at the super- or stripe-anchor region, which is closely associated with hypomethylated DNA regulatory regions that tether super-enhancers to cognate promoters (49). Since the recruitment of cohesin, but not CTCF, to the anchor region (200-kb upstream of *Rag2* gene) was drastically reduced in *R-TEn-E-box-mutant* DP cells, E protein binding to the enhancer regions drives cohesin loading for the local compaction of this gene locus (52). In addition to the critical role of CTCF in TCR/IgH recombination (106–111), CTCF stabilizes long-range promoter-enhancer interactions and controls the cell-to-cell variation in gene expression in mammalian cells (112). With respect to the role of cohesin in *Rag* gene expression, cohesin subunit *Rad21*-deficient thymocytes, in which cohesin was ablated in resting small DP cells by CD4-Cre, showed the impaired secondary distal Jα recombination (102). However, since *Rag* gene SE formation is normally formed in *Rad21*-deficient thymocytes, other molecules may play roles in maintaining *Rag* gene SE formation and their expression after establishment of the SE structure in DP cells (52). Because *Satb1*-deficient DP cells showed a significant reduction in the enhancer-promoter interaction of *Rag* genes, it is reasonable to speculate that cohesin and CTCF drive loop extrusion of the *Rag* gene locus and that Satb1 may act as a chromatin organizer to maintain the SE structure in DP cells (64, 104). Taken together, the data suggest that E protein binding to CREs facilitates the recruitment of cohesin complexes to enhancer and anchor regions to orchestrate adaptive lymphocyte-specific spatial interactions in the *Rag* gene locus to induce robust gene expression (**Figure 3C**). To establish this model, it is necessary to clarify the underlying mechanism of how E proteins recruit the cohesin to enhancer and anchor regions.

Notably, T cell- or B cell-specific enhancers or *Rag1* promoter are accessible at the T- or B-progenitor stage without promoter or enhancer activity, even though both activities depend on E protein binding (52). Furthermore, γδT cell development and TCRβ D-J recombination in DN3a cells were less affected in *R-TEn* deletion mice, while these events were absent in DN3a and pro-B cells obtained from *R1pro-E-box-mutant* mice. Therefore, upon T lineage commitment, E2A binds to both the *Rag* enhancer and promoter independently. The low level of *Rag1* expression induced by *R1pro* activity is sufficient to permit TCRβ D-J and TCRγ δ recombination, which is in line with the fact that TCRγ δ recombination occurs concurrently with TCRβ D-J recombination at the DN2 stage before TCRβ V-DJ recombination (**Figure 3C**) (5, 113). The high level of *Rag* expression induced by enhancers is required for long-range V-(D)J recombination of TCRβ or TCRα (52). From these data, we propose a two-step *Rag* gene regulation model. (1) *Rag1/2* genes are first initiated by E-protein binding to the CREs near exon 1, which induces D-J and TCRγδ recombination. (2) *Rag1/2* gene expression is further enhanced by cell type-specific enhancers to drive their robust expression, which is required for long-range V-(D)J recombination (**Figure 3C**). Considering a previous report showing that the formation of the RAG recombination center (RC) in T cell- or B cell-progenitors/precursors occurs in a developmental stage-specific manner and that RAG1 preferentially binds to TCRβ D-J regions in pro-T and *Igh* Jh regions in pro-B, which have a high level of H3K4 trimethylation (2, 114), we speculate that a low level of RAG1 protein can initially bind to the D-J regions but not to V regions, resulting from the insufficient formation of RAG-RC for V-DJ recombination. Further study is needed to clarify the stepwise effect of *Rag* expression mediated by enhancer-promoter cooperation.

In *R-TEn* deletion mice, the number of γδT cells was moderately reduced in the adult thymus; however, the percentages of Vγ1.1 and Vγ2 in the γδT cell population were not affected (52). Compared with the recombination of TCRβ V-DJ and TCRα V-J, the assembly of TCRγδ occurs within a short range of TCRγ clusters or local segments of TCRδ in the TCRα locus, depending on their own enhancers. The accessibility and recombination of the TCRγ locus rely on IL-7/IL-15-Stat5 signaling (115, 116). Therefore, we speculate that the low RAG1 and RAG2 protein levels are sufficient for RAG-RC to drive local TCR recombination in cooperation with cytokine signaling.

**Figure 4:**
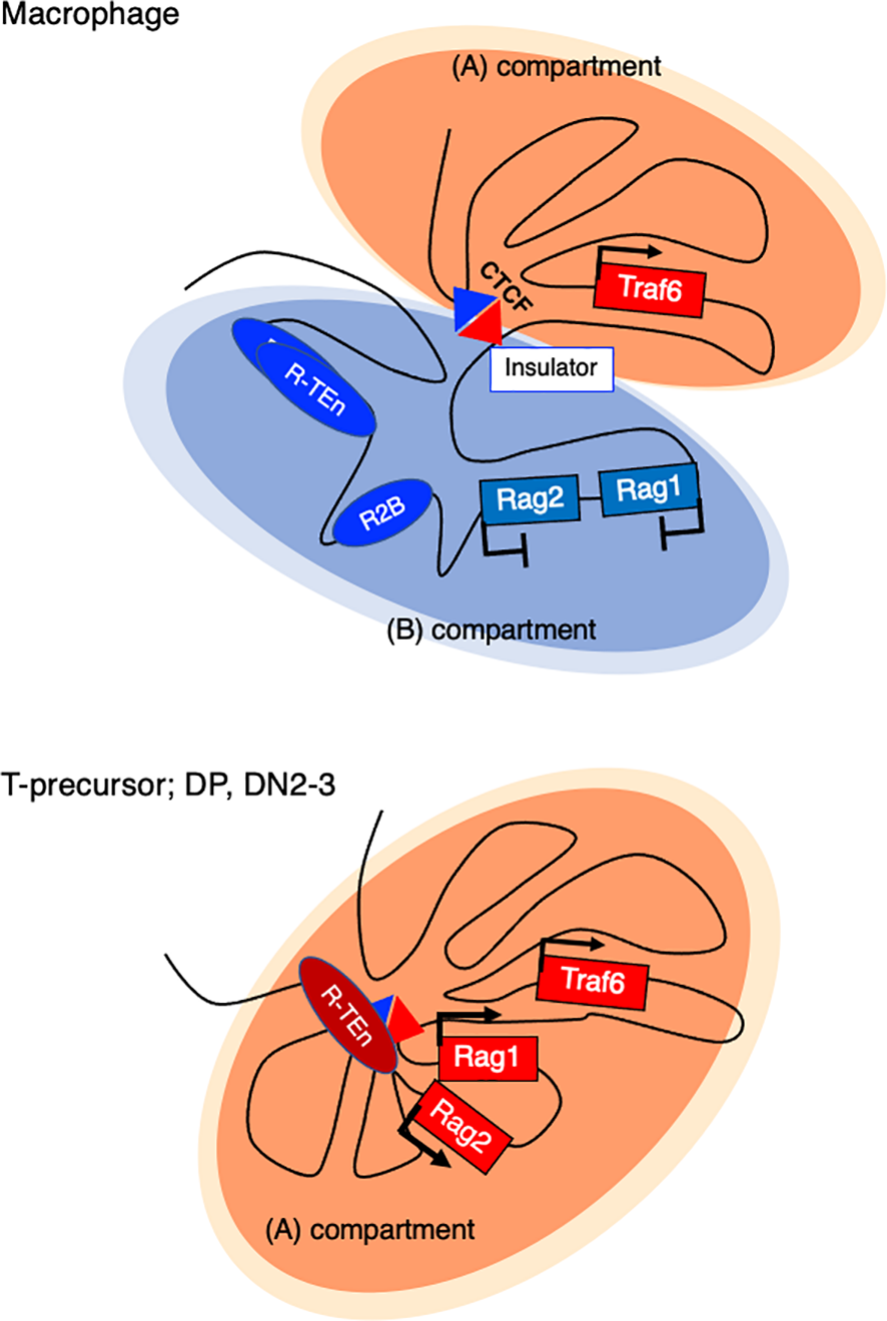
Active and repressive compartmentalization in the *Rag* gene cluster and neighboring *Traf6* gene in a macrophage and T cell precursor. In nonlymphoid cells (Bone marrow derived macrophage (BMDM), a sharp TAD boundary mediated by CTCF forms between *Rag1* and *Traf6*, insulating the entire *Rag* gene locus from the active (A) compartment and sequestering it in a repressive (B) compartment.

How do *Rag1/2* genes get repressed in innate immune cells? In bone marrow-derived macrophages (BMDMs), a sharp TAD boundary between *Rag1* and the neighboring gene *Traf6*, which is generally expressed, is formed to insulate the entire *Rag* gene locus from the active compartment and sequester it in the repressive compartment (**Figure 4**) **(**52). Tight physical regulation illustrates how harmful genome recombinase enzymes are properly regulated at the chromatin architecture level.

## Conclusion

The regulation of *Rag1* and *Rag2* genes is highly conserved, and the expression of these genes is a hallmark of adaptive immunity. Cell type-specific gene expression is driven by the interplay between E2A/E proteins and chromatin interactions. Future experiments are warranted to explore the role of a potential pioneer TF in regulating enhancer activity with other lineage-specific TFs and to understand how these TFs reorganize the 3D genome architecture through the recruitment of the cohesin complex. These findings may have implications for health and immunological disorders.

## Author Contributions

KM and MM wrote the manuscript and figures. All authors contributed to the article and approved the submitted version.

## Funding

This work was funded by the KAKENHI (Grants-in-Aid for Scientific Research) from the MEXT of Japan (19H03487 for MM) and the Mochida Memorial Foundation and the Takeda Science Foundation (MM).

## Conflict of Interest

The authors declare that the research was conducted in the absence of any commercial or financial relationships that could be construed as a potential conflict of interest.
