# Supplementary figures and images for: The Interplay Between Chromatin Architecture and Lineage-Specific Transcription Factors and the Regulation of Rag Gene Expression
Source: Front Immunol. 2021 Mar 16;12:659761. doi: 10.3389/fimmu.2021.659761 (PMC8007930; doi:10.3389/fimmu.2021.659761)

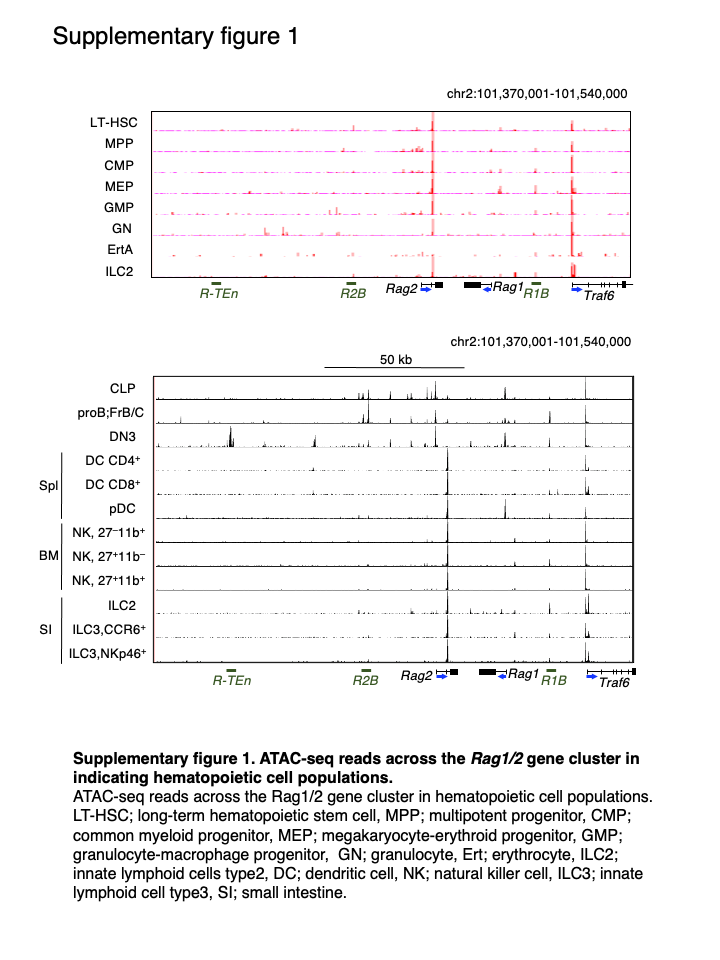

Supplement: Supplementary file 1 [file Image_1.tiff]
